# Understanding Omics Driven Plant Improvement and *de novo* Crop Domestication: Some Examples

**DOI:** 10.3389/fgene.2021.637141

**Published:** 2021-04-06

**Authors:** Rakesh Kumar, Vinay Sharma, Srinivas Suresh, Devade Pandurang Ramrao, Akash Veershetty, Sharan Kumar, Kagolla Priscilla, BhagyaShree Hangargi, Rahul Narasanna, Manish Kumar Pandey, Gajanana Ramachandra Naik, Sherinmol Thomas, Anirudh Kumar

**Affiliations:** ^1^Department of Life Science, Central University of Karnataka, Kalaburagi, India; ^2^International Crops Research Institute for the Semi-Arid Tropics, Hyderabad, India; ^3^Department of Biosciences & Bioengineering, Indian Institute of Technology Bombay, Mumbai, India; ^4^Department of Botany, Indira Gandhi National Tribal University, Amarkantak, India

**Keywords:** omics, metabolomics, *de novo* domestication, crop improvement, domesticated-genes

## Abstract

In the current era, one of biggest challenges is to shorten the breeding cycle for rapid generation of a new crop variety having high yield capacity, disease resistance, high nutrient content, etc. Advances in the “-omics” technology have revolutionized the discovery of genes and bio-molecules with remarkable precision, resulting in significant development of plant-focused metabolic databases and resources. Metabolomics has been widely used in several model plants and crop species to examine metabolic drift and changes in metabolic composition during various developmental stages and in response to stimuli. Over the last few decades, these efforts have resulted in a significantly improved understanding of the metabolic pathways of plants through identification of several unknown intermediates. This has assisted in developing several new metabolically engineered important crops with desirable agronomic traits, and has facilitated the *de novo* domestication of new crops for sustainable agriculture and food security. In this review, we discuss how “omics” technologies, particularly metabolomics, has enhanced our understanding of important traits and allowed speedy domestication of novel crop plants.

## Introduction

**FIGURE 1:**
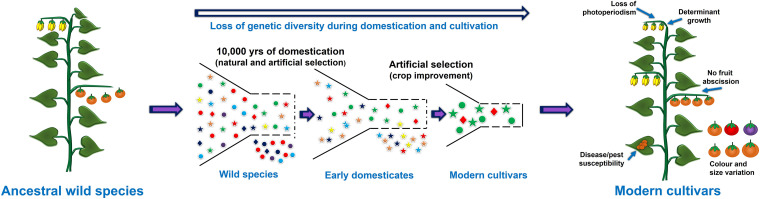
Representation of domestication process and the loss of useful genetic variation due to selective breeding and selection of few alleles.

The process of crop domestication began approximately 12,000 years ago, and was an important milestone during human civilization and led the foundation of modern agriculture. In the 21st century, most of the cultivated crops are domesticated from their wild ancestral progenitors (Meyer et al., 2012). During the domestication process plants were selected based on visible traits guided by needs of the time which led to the selection of only few desired alleles and dilution of the genetic variation present within the crop (Figure 1). For example, in cereals like wheat and rice, traits such as increase in the number of seeds per plant, uniform seed maturation, and non-shattering of seeds were preferred over the size of kernels during early domestication (Si et al., 2016). However, the selection of such traits varies greatly from plant to plant or between crops. For instance, in fleshy fruits or berries such as tomato, eggplant and apple, the size of the fruits and berries were preferred over overall yield (Zhu et al., 2018). Likewise, in tuber producing plants such as potato the tuber size is one of the preferred traits (Fernie and Yan, 2019). Surprisingly, cultivated plant species represent only 250 fully domesticated species among 2500 species, which have undergone the process of domestication, and represent 160 plant families (Smýkal et al., 2018). This proportion is even starker considering the total plant diversity available for the cultivation or those, which are already being cultivated in different places (semi-cultivated species). For example, around 400,000 semi-cultivated plant species are currently known which can be utilized for designing future crops (Smýkal et al., 2018; Fernie and Yan, 2019).

The process of domestication of a species is a very slow and steady process. In fact, the modern cultivars available were generated following a long series of events: (a) Neolithic Revolution, (b) Columbian Exchange, (c) Industrial Revolution, (d) Green Revolution, and (e) Genomic Revolutions (Smýkal et al., 2018). Presently, to feed an ever-growing global population in the face of climate change is challenge for agriculture especially due to reduction of the arable lands due consistent conversion of lands into semi-arid areas and nutrient deficient land along with increase in soil pH or salinity. Therefore, a more rapid method of developing elite climate smart cultivars with desired traits is required. This could be achieved through integrated OMICS approaches, which include genomics, transcriptomics, proteomics, metabolomics and phenomics integrated with bioinformatics analyses (Kumar et al., 2017, 2018; Sharma et al., 2021). Plant OMICs based research have played very important role in deciphering metabolic pathways and their molecular regulators, which govern key traits and several plant developmental processes (Kumar et al., 2017; Razzaq et al., 2019). In the past decade there has been a significant progress in the field of both sequencing (van Dijk et al., 2018; Kumar et al., 2020; Schmidt et al., 2020) and analytical methods for the detection of molecules (Ren et al., 2018; Gilmore et al., 2019; Macklin et al., 2020), which has not only improved detection throughput but also the accuracy and the sensitivity (Kumar et al., 2017; Chiang et al., 2018; Qi et al., 2019).

In the past, for the selection of traits breeding programs involved phenotypic selection of plants (which are guided by metabolic pathways) (Kiszonas and Morris, 2018). For instance, during the Green Revolution (from 1960 to 1980), development of semi-dwarf high yielding varieties of rice and wheat involved phenotypic selections of improved lines which actually involved selection of gibberellic acid pathway genes including the *GA20 oxidase* and *DELLA* protein encoding genes (Silverstone and Sun, 2000). In fact, most of the traits, which were targeted for the Green Revolution, are controlled by one or more metabolic pathways. Therefore, precise editing of these metabolic pathways can help in the development of varieties in a very short time (Rodríguez-Leal et al., 2017; Zhang Y. et al., 2018; Fernie and Yan, 2019). Previously, most of the reviews on plant omics have focused on the instrumentation involved and results obtained by different researchers (Kumar et al., 2017; Mangul et al., 2019; Misra et al., 2019; Tang and Aristilde, 2020). In this review, we represent how this omics knowledge can be utilized for development of improved cultivars by targeting metabolic pathways and also emphasize the use of this information for *de novo* domestication of wild ancestral species for sustainable food security.

## Role of Omics Data in Understanding Plant Traits

Genomics plays an important role in the identification of quantitative trait loci (QTLs) and genes controlling important traits, particularly in domesticated crops (Fernie and Yan, 2019). Moving forward, great insights have been gleaned from genome sequencing and re-sequencing programs examining wild ancestral species of domesticated crops (Unamba et al., 2015). In plant genomics, Next Generation Sequencing (NGS) has played a very important role and provided opportunities in the field of functional genomics due to the availability of reference genomes for several model and crop plant species. These resources combined with high quality re-sequencing offers huge potential for discovery of causal genes and mechanisms associated with the key agronomic traits (Thudi et al., 2016; Chen et al., 2019; Varshney et al., 2019). Re-sequencing also enriched the availability of SNPs data and can be utilized for genomics-based studies such as GWAS (genome wide association study) and QTL-seq (Kumar et al., 2020), both of which are useful tools for trait mapping (Rivas et al., 2011; Zhu et al., 2011; Zhang et al., 2021). With the advent of these technologies combined with advances in metabolomics such as integration of GWAS with metabolomics efficient means for dissecting underlying molecular mechanisms involved in the growth and development are available (Table 1; Fang and Luo, 2019).

**TABLE 1:** List of selected studies involved mQTL and mGWAS approach.

Plant	Population/accessions	Approach	Tissue	Study	Significant outcome	References
Apple (*Malus domestica*)	Prima × Fiesta	LC-MS	Fruit	mQTL	Identified 669 mQTLs, includes a major mQTL hotspot on LG16 contains gene *leucoanthocyanidin reductase* belong to the phenylpropanoid pathway.	Khan et al., 2012
*Arabidopsis thaliana*	Col-0 × C24 (RIL), ILs	GC-MS	Leaf	mQTL	Identified 385 mQTL for 136 metabolites	Lisec et al., 2009
	*A. thaliana* accessions	LC-MS	Leaf	mGWAS	Identification of 123 mQTL and 70 candidate genes	Wu et al., 2018
	314 natural accessions	GC-MS	Leaf	mGWAS	Identify two candidate genes (AT5G53120 and AT4G39660) involved in the β-alanine metabolic pathway	Wu et al., 2016
	Bay × Sha (RIL)	GC-MS	Leaf	mQTL	Identified 11 mQTL clusters linked to the plant central metabolism.	Rowe et al., 2008
	RILs and ILs	GC-MS	Seedling	mQTL	Identified 153 QTLs for augmented additive (Z1) and 83 QTL for dominance effects (Z2) in RIL	Lisec et al., 2009
	96 accessions	HPLC-DAD	Leaf	mGWAS	Identified two major QTLs controlling glucosinolate variation; and *AOP* and *MAM as* candidate genes	Chan et al., 2010
	313-ecotype association panel	LC-MS	Seed	mGWAS	Identified two significant associated genomic regions (One region is linked with serine-related trait and second region is linked with four histidine-related traits)	Angelovici et al., 2017
	Col-0 × C24	GC-MS	Seed	mQTL	Identified 786 mQTLs and candidate genes including *bZIP10* as regulator of seed metabolism	Knoch et al., 2017
Barley (*Hordeum vulgare*)	Diverse set of barley accessions	LC-MS	Flag leaf	mGWAS	Reported three mQTLs for metabolites (γ-tocopherol, glutathione, and succinate content) involved in antioxidative defense	Templer et al., 2017
	Maresi** ×** CamB (RIL)	LC-MS	Leaf	mQTL	Identified 138 mQTLs for 98 traits. Annotation of mQTL identified genomic region with stress response related genes	Piasecka et al., 2017
	Qingke and barley accessions including wild	LC-MS	Leaf and Seed	mGWAS	Identified 90 significant mGWAS loci for variation of phenylpropanoid content	Zeng et al., 2020
Blueberry (*Cyanococcus*)	886 blueberry genotypes	GC-MS	Fruits	mGWAS	Identified 519 significant SNPs linked to 11 volatile organic compounds	Ferrão et al., 2020
Maize (*Zea mays* L.)	By804 × B73 (RIL)	GC-MS	Seedling, Leaf, Kernel	mQTL	Detected 297 QTL and candidate genes to the amino acid biosynthetic and catabolic pathways, tricarboxylic acid cycle and carbohydrate metabolism	Wen et al., 2015
	Inbred lines	GC-MS	Leaf	mGWAS	Identified 26 distinct metabolites strong associations with leaf complex trait such as dry mass, lignin composition etc.	Riedelsheimer et al., 2012
	Inbred lines	HPLC	Grain	mGWAS	Identified *ZmVTE4* haplotype and three new gene targets for increasing antioxidant and vitamin E levels. Also identified two additional genes, *ZmHGGT1* and one prephenate dehydratase parolog that modestly contribute to tocotrienol variation	Lipka et al., 2013
	Inbred lines	UP-LC	Kernel	mGWAS	Identified 74 loci functionally associated with kernel oil content and fatty acid composition; Also identified genes involved in oil biosynthesis (*DGAT1-2*, *FATB* and *FAD2*), members of the oil metabolic pathway (*FAD2*, *LCACS*, *ACP*, and *COPII*) and one transcription factor (*WRI1a*)	Li et al., 2013
	Inbred lines	HPLC	Kernel	mGWAS	Nine carotenoid compounds measured in grain samples, the most abundant was zeaxanthin; Identified 58 candidate genes involved in biosynthesis and retention of carotenoids in maize.	Owens et al., 2014
	Inbred lines and RIL population	LC-MS	Mature Kernel	mGWAS	Identified 1,459 significant locus–trait associations across three environments through metabolite-based genome-wide association mapping, identified potential causal variants for five candidate genes involved in metabolic traits	Wen et al., 2014
	Inbred diversity panel	LC-MS	Kernel	mGWAS	Identified 19 modules which shows significant associations with genetic control of biochemical networks within the kernel.	Shen et al., 2013
	513 diverse inbred lines association panel	GC-MS	Seedling, Leaf, Kernel	mGWAS	Identified 153 significant loci linked to primary metabolism	Wen et al., 2018
Potato (*Solanum tuberosum*)	Diversity panel	LC-MS	Tuber	mGWAS	Identified 472 features in which significant SNPs have been associated to glycoalkaloids (α-chaconine, β-chaconine, and α-solamarine) reported on chromosomes 2, 7, and 8	Levina et al., 2020
	C (*S. phureja* × S. tuberosum) × E (*S. vernei* × *S. tuberosum*)	GC-MS	Tuber	mQTL	Identified 87 mQTLs associated to primary metabolism	Carreno-Quintero et al., 2012
Rapeseed (*Brassica napus*)	EXPRESS × SWU07 (DH)	NIRS	Seed	mQTL	Identified four QTLs for Glucosinolates content between	He et al., 2018
	Tapidor × Ningyou7 (DH)	HPLC	Leaf and Seed	mQTL	105 mQTLs related to glucosinolate biosynthesis in rapeseed seed and leaves have been observed	Feng et al., 2012
Rice (*Oryza sativa*)	ZS97 × MH63 (RIL)	LC-MS	Flag leaf, germinating Seed	mQTL	Identified 1,884 mQTLs in flag leaf and 937 mQTLs in germinating seed samples	Gong et al., 2013
	Sasanishiki × Habatak (BIL)	GC-MS, LC-MS, CE-MS	Seed	mQTL	Identified 802 mQTLs for 759 metabolic traits; including mQTL hotspot on chromosome 3 regulating amino acids content	Matsuda et al., 2012
	Landraces accessions and subpopulations rice subspecies *indica* and *japonica*	LC-MS	Five-leaf stage	mGWAS	Identified 36 candidate genes controlling metabolites level and nutritional composition	Chen et al., 2014
	Landraces accessions	LC-MS	Leaf/seedling	mGWAS	Identified 323 associations, demonstrating that phytochemicals produced in rice cultivars are diverse	Matsuda et al., 2015
	Landraces and elite varieties of indica and japonica	LC-MS	Grains	mGWAS	More than 30 candidate genes were identified, associated with metabolic and/or morphological traits.	Chen et al., 2016
	156 Landrace	LC-MS	Leaf/root and other tissue parts of rice	mGWAS	Identified two *spermidine hydroxyl-cinnamoyltransferases* (Os12g27220 and Os12g27254) that might underlie the natural variation levels of spermidine conjugates in rice	Dong and Wang, 2015
	ZS97 × MH63 (RIL)	LC-MS	Leaf and Seed	mQTL	Provided over 2,800 highly resolved metabolic quantitative trait loci for 900 metabolites; associated 24 candidate genes to various metabolic quantitative trait loci by data mining, including ones regulating important morphological traits and bio-logical processes	Gong et al., 2013
	Three CSSL populations (N/Z, M/Z, and A/Z)	LC-MS	Flag leaf	mQTL	Identified 1,587 mQTL, of which 684 in (A/Z), 479 in (M/Z), and 722 in(N/Z) have been detected among three CSSL population	Chen et al., 2018
	Lemont × Teqing (RIL)	GC-MS	Leaf	mQTL	Identified two mQTL hotspots which have opposing effects on carbon and nitrogen rich metabolites, and regulate carbon and nitrogen partitioning.	Li et al., 2016
Strawberry (*Fragaria* × *ananassa*)	*F.* x *ananassa* 232 × 1392 (F1)	LC-MS	Fruit	mQTL	Reported 309 mQTLs for 77 polar secondary metabolites.	Pott et al., 2020
	232 × 1392 (F1)	GC-MS	Fruit	mQTL	Reported 133 unique mQTLs for 44 traits with PVE% range from 9.6% to 46.1%. RNA seq analysis identified candidate gene *Mannose-6-phosphate isomerase* responsible for natural variation in L-ascorbic acid in fruit	Vallarino et al., 2019
Tomato (*Solanum lycopersicum*)	Introgression lines	LC-MS	Fruit	mQTL	Detected 216 canalization metabolite quantitative trait loci (cmQTLs) for secondary metabolites and 93 cmQTLfor primary metabolites.	Alseekh et al., 2017
	Introgression lines	UPLC	Fruit	mQTL	Identified 679 mQTLs for primary metabolites and secondary metabolites	Alseekh et al., 2015
	Introgression lines	GC-MS	Seed	mQTL	Identified 46 mQTLs in IL population and propose post transcriptional regulation	Toubiana et al., 2012
	Tomato accessions including wild	GC-MS	Fruit	mGWAS	Identified a total 44 loci associated with 19 traits, including sucrose, ascorbate, malate and citrate levels.	Sauvage et al., 2014
	Tomato accessions including wild	GC-MS	Fruit	mGWAS	Identified 388 suggestive association loci (including 126 significant loci) for 92 metabolic traits including nutrition and flavor-related loci by genome-wide association study	Ye et al., 2019
	IL12-3 × M82	LC-MS	Fruit and leaf	mQTL	Reported 1528 mQTLs in fruit and 428 mQTL in leaf; Major mQTL involved in the regulation of diacylglycerols and triacylglycerols have been detected on chromosome 12	Garbowicz et al., 2018
	76 ILs + recurrent parent M82	LC-MS	Seed	mQTL	Identified 338 mQTL for flavonoids, steroidal glycoalkaloids, and specialized metabolites content	Alseekh et al., 2020
	IL4-4 × M82	GC-MS, HPLC, LC-MS	Fruit	mQTL	Identified 72 mQTL, where major mQTLs linked to twenty genes which have a broad effect on several metabolic pathways.	Liu et al., 2016
	ILs	GC-MS	Fruit	mQTL	Reported 889 fruit traits related mQTLs and 326 yield-related traits mQTLs	Schauer et al., 2006
	IL and heterozygous ILH	GC-MS	Fruit	mQTL	Identified 332 putative mQTL associated with metabolite accumulation (174 mQTLs is dominantly inherited, 61 mQTLs is additively inherited and 80 mQTLs is recessively inherited and negligible number of mQTL showing the feature of over dominant inheritance)	Schauer et al., 2008
	*S. lycopersicum* (M82) × *S. pennellii* Ils	GC-MS, LC-MS, HPLC-PDA, NMR	Fruit	mQTL	Detected mQTL for carotenoids and tocopherols	Perez-Fons et al., 2014
Wheat (*Triticum aestivum*)	KN9204 × J411 (RIL)	LC-MS	Kernel	mQTL	Identified 1005 mQTLs and 24 genes candidate gene related to grain related traits	Shi et al., 2020
	Excalibur × Kukri (DH)	LC-MS	Flag leaf	mQTL	Identified mQTLs for 238 metabolites across 159 intervals on genetic map; two mQTLs on chromosome 7A coordinating the genetic control of various metabolites	Hill et al., 2015
	Winter elite lines (135)	GC-MS, LC-MS	Flag leaf	mGWAS	Identified significant associations 17 SNPs with six metabolic traits, namely oxalic acid, ornithine, L-arginine, pentose alcohol III, L-tyrosine, and a sugar oligomer (oligo II)	Matros et al., 2017
	Natural accessions	LC-MS	Mature seeds	mGWAS	A total of 1098 mGWAS associations were detected with large effects, within which 26 candidate genes for flavonoid decoration pathway	Chen et al., 2020
	Doubled haploid lines	GC-MS	Flag leaf	mQTL	Identified 112 mQTLs for 95 metabolites, of which 43 are known compounds	Hill et al., 2013

### Sequencing and QTL-seq Based Trait Discovery

Presently, QTL-seq is one of the most successful approach developed for the gene discovery and trait dissection (Kumar et al., 2020; Pandey et al., 2020). This approach offers preliminary idea for positional cloning for linked genetic factors or genes, and it has excellent success in marker-assisted selection for crop improvement programs (Xu F. et al., 2015). With the advancements in NGS technologies new approaches like quantitative trait locus sequencing (QTL-seq) has been utilized for exploring rapid QTL or gene identification (Takagi et al., 2013). In QTL-seq approach, the extreme highest and lowest genotypes are selected from the mapping population for target traits, followed by mixing an equal amount of DNA from each bulk to build up two extreme bulk (High bulk and low bulk). Then, each bulk is sequenced and assembled and gene annotation is performed. This approach combined with Bulked segregant analysis, accompanied by whole genome re-sequencing technologies, is more effective and capable than the previous QTL detection methods (Takagi et al., 2013). Utilizing QTL-seq approach several QTLs and genes for different rice phenotypes (Takagi et al., 2013; Daware et al., 2016; Ogiso-Tanaka et al., 2017; Yang et al., 2017; Kadambari et al., 2018; Qin et al., 2018; Bommisetty et al., 2020; Nubankoh et al., 2020), soybean (Song et al., 2017; Zhang X. et al., 2018), chickpea (Singh et al., 2016; Deokar et al., 2019), tomato (Illa-Berenguer et al., 2015), groundnut (Kumar et al., 2020; Luo et al., 2019; Zhao et al., 2020), have been effectively identified. This approach has also been deployed across in several crops due to its inherent ability to understand both qualitative and quantitative traits (Table 2). For instance, Kumar et al. (2020) identified the role of two genes *RING-H2 finger protein* and *zeaxanthin epoxidase* in fresh seed dormancy in groundnut; both genes are known to control abscisic acid (ABA) accumulation. Very recently, Ramos et al. (2020) identified three QTLs (genomic regions) *viz* QtlPC-C04, QtlPC-C11 and QtlPC-C14 (linked to genes *R1R2R3*) associated with resistance to *Phytophthora capsici* Leonian which causes crown rot in squash (*Cucurbita moschata*). The most significant benefit of whole genome sequencing is that it allows the identification of causative mutations in target chromosomal regions. Additionally, this method identifies molecular markers which are located inside the harboring chromosomal region that can also be used to narrow down the genomic region which will help in mining the trait linked genes.

**TABLE 2:** List of important QTL-seq studies in crop plants.

Crop	Population	Target Trait	QTL/Gene mapped	References
*Oryza sativa*	IR 64 × Sonasal	Grain Weight	Two genes LOC_Os05g15880 (glycosyl hydrolase) and LOC_Os05g18604 (serine carboxypeptidase)	Daware et al., 2016
	Nipponbare × BIL-55	Late heading under short-day conditions	Zinc finger B-box domain containing protein (Os04t0540200-01), WD40-repeat-domain–containing proteins (Os04t0555500-01, Os04t0555600-01, Os04t0564700-01), flowering locus D (Os04t0560300-01), CCAAT-binding-domain–containing protein (Os06t0498450-00)	Ogiso-Tanaka et al., 2017
	H12-29 × FH212	Grain Length and Weight	*qTGW5.3* (1.13 Mb)	Yaobin et al., 2018
	LND384 × INRC10192	Plant height	*asd1* (67.51 Kb)	Kadambari et al., 2018
	M9962 × Sinlek	Spikelet fertility	*qSF1, qSF2, and qSF3 (LOC_Os01g59420, LOC_Os02g31910, LOC_Os02g32080, LOC_Os03g50730)*	Nubankoh et al., 2020
	BPT5204 × MTU3626	Grain weight	*qGW8* (LOC_Os08g01490 (Cytochrome P450), and LOC_Os08g01680 (WD domain, G-beta repeat domain containing protein)	Bommisetty et al., 2020
*Triticum aestivum*	GY448 × GY115	Awnless trait	*Qal.nwipb-5AL* (25-bp indel in *B1* gene promoter region)	Wang et al., 2021
*Zea mays*	CMS-C lines × A619	Fertility Restoration	*qRf8-1* (17.93-Mb)	Zheng et al., 2020
*Brassica napus*	Huyou19 × Purler	Branch angle	Branch angle 1 (BnaA0639380D, a homolog of AtYUCCA6)	Wang et al., 2016
	Cabriolet × Darmor	Vernalization	FLOWERING LOCUS C (*BnaFLC.A02*) and FLOWERING LOCUS T (*BnaFT.A02*)	Tudor et al., 2020
*Brassica rapa*	Zicaitai × Caixin	Purple Trait	*BrMYBL2.1* gene	Zhang X. et al., 2020
*Glycine max*	Zhonghuang × Jiyu 102	Seed cotyledon color	qCC1 (30.7-kb) and qCC2 (67.7-kb)	Song et al., 2017
	CSSL3228 × NN1138–2	Plant height	Glyma.13 g249400 candidate gene	Zhang X. et al., 2018
*Arachis hypogaea*	ZH8 × ZH9	Testa color	*AhTc1*, encoding a R2R3-MYB transcription factor	Zhao et al., 2020
	ICGV 00350 × ICGV 97045	Fresh seed dormancy	RING-H2 finger protein and zeaxanthin epoxidase	Kumar et al., 2020
	Yuanza 9102 × Xuzhou 68-4	Shelling percentage	Nine candidate genes in 10 SNPs	Luo et al., 2019
*Cicer arietinum*	ICC 4958 × ICC 1882	100-seed weight	Two genes *Ca_0436* and *Ca_04607*	Singh et al., 2016
	ICCV 96029 × CDC Frontier and ICCV 96029 × Amit	Ascochyta blight	Six candidate genes on chromosomes Ca2 and Ca4	Deokar et al., 2019
*Solanum lycopersicum*	Three populations (12S139, 12S143 and 12S75)	Fruit weight and locule number	Three fruit weight (*fw1.1*, *fw3.3*, *fw11.2*) and one locule number (*lcn6.1*) QTLs	Illa-Berenguer et al., 2015
*Cucumis melo*	MR-1 × M1-32	Stigma Color	GS8.1 (268 kb) MELO3C003149, MELO3C003158, and MELO3C003165	Qiao et al., 2021
*Cucumis sativus*	PM-R × PM-S	Powdery mildew resistance	*Two QTLs pm5.2* and *pm6.1* (*CsGy5G015660*)	Zhang et al., 2021

### RNA-seq Based Trait Discovery

Advances in RNA sequencing (RNA-seq) technologies and approaches have made significant impact toward trait discovery, and have enabled plant developmental studies characterizing expression patterns of all the functional genes as well as regulatory RNAs (Nayak et al., 2019). RNA-seq is a more robust approach for precise measurement of transcripts and has been widely used for transcript profiling in several plant species (Cloonan et al., 2008; Wang et al., 2009). This data is vital for improving genome annotations, and offers precise gene position information for functional characterization and genome editing. The RNA-seq approach has been deployed for molecular characterization of several important agronomic traits such as seed size (Wan et al., 2017), seed coat color (Wan et al., 2018), seed coat cracking (Wan et al., 2016), seed and bud dormancy (Qi et al., 2015; Zhu et al., 2015; Khalil-Ur-Rehman et al., 2017), fatty acid biosynthesis and oil quality (Nayak et al., 2019), nutritional quality traits (Reddy and Ulaganathan, 2015), etc., which can offer precise gene information for developing designer crops for future. Also, RNA-seq have been used to understand the molecular mechanisms associated with salt tolerance in rice (Zhou et al., 2016; Lei et al., 2020); Chinese rye grass (Sun et al., 2013), and maize (Liang and Schnable, 2016). In wheat, RNA-seq study reported the drought responsive molecular pathways along with key candidate genes and molecular markers in the root tissue (Iquebal et al., 2019). RNA-seq has also been shown to be highly useful in combination with other -omics methods for gene discovery and pathway investigations.

### Proteomics Enabled Genetic Trait Understanding

Knowledge of proteomics is being used to map the translated genes and loci controlling the expression of respective genes. It helps in identification of proteins responsible for bringing intricate phenotypic variations. High throughput proteomics has gone beyond the identification of individual proteins, to quantitative profiling, post translational modification studies, signaling, protein–protein interaction and many more areas. Photosynthesis plays major role in biomass production and yield. Change in protein profile studies was performed in chlorophyll deficient *Brassica napus* leaves which established the relationship between chlorophyll biosynthesis and photosynthesis (Chu et al., 2015). Xylem sap proteomics has revealed several insights related to cell wall formation (Zhang M. et al., 2014), leaf senescence (Wang et al., 2012) biotic and abiotic stress response (Alvarez et al., 2008; González et al., 2012), cell to cell communication (Agrawal et al., 2010), and root–shoot communication (Krishnan et al., 2011). The enhanced level of redox proteins, stress and defense related proteins, calcium ion regulation proteins, signaling G-protein and RNA metabolism related proteins were induced in phloem sap study. Recently, proteomics study revealed that low light stress obstructs carbon fixation and *OsGAPB* overexpression augment tolerance to low light stress conceivably by increasing CO_2_ assimilation and chlorophyll accumulation in rice (Liu et al., 2020). Interestingly, simultaneous upregulation of both biotic and abiotic stress responsive protein has been observed during bacterial blight infection in rice, which indicate the activation of common pathway (Kumar et al., 2015). Whereas in case of rice-*M. oryzae* interaction PBZ1, OsPR-10, SalT, Glu1, Glu2, and TLP proteins were up-regulated (Kim et al., 2004). iTRAQ proteomics study of *Oryza officinalis* provided evidences that proteins related to biosynthesis of secondary metabolites and carbon metabolism were mostly enriched after planthopper infestation (Zhang et al., 2019c). Several proteomics and transcriptomics study conducted on seed dormancy study revealed the important role of antioxidant mechanism, protein thiol and redox regulation along with hormonal signaling in rice, wheat and barley (Hu et al., 2015). Mass spectrometry (MS) based proteomics study demonstrated the cultivar specific induction of proteins in salt stress condition such as glutathione-based detoxification of ROS was highly induced in tolerant variety whereas proteins involved in iron uptakes were more expressed in salt sensitive variety in barley (Witzel et al., 2009). Similarly, the role of *OsCYP2* in moderating the antioxidant enzymes was established in transgenic rice overexpressing cyclophilin during salt stress (Ruan et al., 2011). Seed proteomics of various developmental stages during different stresses have revealed the process involved in seed dormancy, seed germination, and seed development (Finnie et al., 2011). Proteomics related to environmental changes and abiotic stress focused on water supply responsive proteins in wheat against drought, high temperature, low temperature, frost, salt and heavy metals have been carried out (Yang et al., 2011; Han et al., 2013; Kosová et al., 2013; Alvarez et al., 2014; Capriotti et al., 2014; Kang et al., 2015). These studies offered novel insights and better understanding of crop physiology and metabolism during various kinds of stresses.

### Metabolomics Based Trait Understanding

Holistic metabolomics based to study trails in plants were started late, particularly this approach was started through the introduction of untargeted metabolome detection (Alonso et al., 2015). Several studies have been reported where a particular metabolic pathways have been mapped (Sharma et al., 2021). For instance, the substantial changes in the various phytohormones was investigated in poplar leaf (Novák et al., 2008), rice various aerial organs (Kojima et al., 2009), rosemary leaves et al. (Müller and Munné-Bosch, 2011), Arabidopsis developing seeds (Kanno et al., 2010), strawberry fruits (Gu et al., 2019), potato tuber (Peivastegan et al., 2019), wheat developing seeds (Matsuura et al., 2019), watermelon fruit (Kojima et al., 2021), etc. The targeted approach has been also applied to explore the carotenoid pathway (Fernandez-Orozco et al., 2013; Kim et al., 2016; Mibei et al., 2017; Yoo et al., 2017; Price et al., 2018; Di Lena et al., 2019), flavonoid pathways (Karimi et al., 2011; Dong X. et al., 2014; Torres et al., 2019), amino acids (Torres et al., 2019; Praveen et al., 2020), and fatty acids (Talebi et al., 2013; Vidigal et al., 2018). Such profiling studies has helped in improving several important traits in plants by targeting specific pathways. Almost 10 years back Liu et al. (2011) targeted fatty acids biosynthesis pathways for enhancing biofuel production. Very recently and *fatty acid desaturase 2* was targeted in several crops such as canola (Okuzaki et al., 2018), peanut (Yuan et al., 2019), rice (Abe et al., 2018), false flax (Morineau et al., 2017), and Soybean (Wu et al., 2020), for enhanced production of oleic acid (C18:1), respectively.

Several un-targeted metabolomics approach has been utilized to target multiple class of metabolites (amines, sugars, organic acids, etc.) from a sample extracted from various tissues of the model and crop plants such as Arabidopsis, apple, groundnut, kiwi fruit, alpine bird’s-foot-trefoil, strawberry, grapes, mango, maize, medicago, orange, pear, sunflower, soybean, tomato, rice, white lupin, etc. (Sharma et al., 2021). Now, the targeted and un-targeted metabolomics approach have been coupled with genomics data for carrying out metabolomics-based quantitative trait locus (mQTL) and metabolic genome-wide association studies (mGWAS) studies (Wen et al., 2015; Chen et al., 2016); which simultaneously identifies the genomic region, causal genes and key metabolites and associated metabolic pathways that govern particular trait in plants. Recently, Li K. et al. (2019) identified 65 primary metabolites *viz* 22 amino acids, 21 organic acids, 12 sugars, four amines and six miscellaneous metabolites in the leaf of teosinte (an ancestor of maize) and identifies advantageous genes present in the wild relative associated with grain yield and shape trait in maize. In tomato, for one of the important trait accumulation of secondary metabolite in fruit was analyzed, and reported several subset of mQTLs- including mQTLs associated with acyl-sugar, hydroxycinnamates, naringenin chalcone, and a range of glycoalkaloids (Alseekh et al., 2015). Likewise, there are several studies which identified key genomic regions, candidate genes and mQTLs related to important traits through mQTL and mGWAS based studies including some domesticated traits, this was extensively reviewed by Sharma et al. (2021).

Previously, a combined transcriptome, proteome and metabolomics approach was used to investigate the ripening process with a final aim of extending tomato fruit shelf life (Osorio et al., 2011). This study showed a strong relationship between metabolites and their associated transcripts controlling ripening such as sugars, organic acids, and cell wall metabolism pathways. Similar studies have been done for banana which led to identification of genes including *ERF1B*, *fructose-1,6-bisphosphatase* and *polygalacturonase* as key regulators of pulp ripening (Li T. et al., 2019). Recently, a combined transcriptome and metabolome study was deployed to study the molecular aspects of resistance and the interaction between *Trichoderma harzianum* strain T22 with tomato during defense responses against aphids (Coppola et al., 2019). This study demonstrated the importance of plant transcription factor families such as ZIP, MYB, NAC, AP2-ERF, and WRKY in biotic stress resistance. These examples show the potential of the -omics studies, working in tandem to characterize complex molecular interactions. These data have been used for the development of several gene expression/proteome/metabolome atlases to facilitate omics-driven crop improvement (Table 3).

**TABLE 3:** List of gene-expression, proteome and metabolome atlas developed in plant.

Plant name	Scientific name	Tissue/cell type	Gene/Proteins/Metabolites	Citations	DOI
*Gene expression atlas*			*Genes*		
Chickpea	*Cicer arietinum*	27	15,947	Kudapa et al., 2018	10.1111/pce.13210
Peanut	*Arachis hypogaea*	19	NA	Sinha et al., 2020	10.1111/pbi.13374
Soybean	*Glycine max*	14	66210	Libault et al., 2010 Severin et al., 2010	10.1111/j.1365-313X.2010.04222.x 10.1186/1471-2229-10-160
Wheat	*Triticum aestivum*	32	94,114	International Wheat Genome Sequencing Consortium (IWGSC)	10.1126/science.aar7191
Rice	*Oryza sativa*	40	∼30,000	Jiao et al., 2009	10.1038/ng.282
Maize	*Zea may*	11	22,151	Sekhon et al., 2013	10.1371/journal.pone.0061005
Bryophyte	*Physcomitrella patens*	10	∼32500	Ortiz-Ramírez et al., 2016	10.1016/j.molp.2015.12.002
*Proteome atlas*			*Proteins*		
Arabidopsis	*Arabidopsis thaliana*	9	13,029	Baerenfaller et al., 2008	10.1126/science.1157956
Rice	*Oryza sativa*	3	2,528	Koller et al., 2002	10.1073/pnas.172183199
Wheat	*Triticum aestivum*	24	46,016	Duncan et al., 2017	10.1111/tpj.13402
*Metabolome atlas*					
Arabidopsis	*Arabidopsis thaliana*			Wu et al., 2018	10.1016/j.molp.2017.08.012

## Molecular Regulations of Domestication Related Traits: Selected Examples

Over the past two decades the molecular regulation and the associated metabolic pathways of several agronomic traits has been revealed because of intensive research and the deployment of omics tools (Table 4). For the major domesticated traits their associated genes pathways have been linked with metabolic networks; however, more focused research is required to understand their specific role in particular metabolic pathways. Here, we review progress in omics-based investigations of several important domestications related traits.

**TABLE 4:** List of genes domesticated in the past and associated metabolic pathways.

Crops	Traits	Domesticated Genes	Involvement in the metabolic pathways	References
Rice	Plant architecture	*sd1*	Encodes gibberellin 20-oxidase (Gibberellin pathway gene)	Spielmeyer et al., 2002
	Seed shattering	*sh4*	Abscisic acid response elements (ABREs) have been identified which is involved in ABA hormone signal pathways	Yan et al., 2015
		*qSH1*	APETALA2-like transcription factor SUPERNUMERARY BRACT (SNP) positively regulates the expression of two rice genes, *qSH1* and *SH5* (SNB-involved regulating pathway)	Jiang et al., 2019
	Awn	*LABA1 / An-2*	*An-2* encodes a cytokinin synthesis enzyme that modulates awn length	Gu et al., 2015; Hua et al., 2015
		*qAWNL2*	N.A	Amarasinghe et al., 2020
	Seed and hull color	*Rc and Rd*	Involved in proanthocyanidin synthesis via the flavonoid pathway	Sweeney et al., 2006; Furukawa et al., 2007
	Seed dormancy	*Sdr4*	Zinc finger protein, *OsVP1* activates *Sdr4* expression to control rice seed dormancy via the ABA signaling pathway	Sugimoto et al., 2010; Chen et al., 2020
	Grain size	*qSW5/GW5*	GW5/ *qSW5* involved in brassinosteroid signaling pathway to regulate grain width and weight (Novel nuclear protein)	Shomura et al., 2008; Weng et al., 2008; Liu et al., 2017
		*Gn1a*	Encodes cytokinin oxidase	Ashikari et al., 2005
Maize	Plant architecture	*tb1 (teosinte branched1)*	Two maize mutants, *teosinte branched1* (*tb1*) and *grassy tillers1* (*gt1*), have been shown affected in the regulation of auxin biosynthesis pathway	Doebley et al., 1997; Whipple et al., 2011
		*br2*	Gene modulates the transport of auxin	Zhang et al., 2019b
	Inflorescence architecture	*ra1 (ramosa1), Tga1*	R*A1* involved in the *ramosa* pathway (Transcription factor)	Sigmon and Vollbrecht, 2010
	Grain filling	*ZmSWEET4c*	Hexose transporter, SWEET4c is important for the Glc to the starch biosynthesis in the endosperm during embryogenesis	Sosso et al., 2015
Wheat	Vernalization	*Vrn2 (ZCCT1 and ZCCT1)*	Likely to coordinate with GA, ABA, cytokinin, and JA signaling pathway	Yan et al., 2004; Deng et al., 2015
		*Vrn1*	Central gene in vernalization pathway similar to *APETALA* of *Arabidopsis*. Linked with GA, ABA, Cytokinin, and JA signaling pathway	Yan et al., 2003; Deng et al., 2015
	Free threshing	*Q* and *homeologs*	Involved in secondary cell wall synthesis pathways and regulation of chemical composition of glumes	Zhang Z. et al., 2020
	Plant architecture	*Rht-1*	Repressor of gibberellic acid pathway	Thomas, 2017
Sorghum	Plant architecture	*dw3*	Gene modulates the transport of auxin	Multani et al., 2003
	Grain pigmentation	*Tannin1 (Tan 1)*	*Tan1* gene, encoding a WD40 protein, that regulate the tannin biosynthesis	Wu et al., 2012
Barley	Inflorescence architecture	*Vrs2*	*Vrs2* expression influences the expression of genes that regulate biosynthesis and metabolism of auxin and cytokinin (Transcription factor, HD-ZIP)	Komatsuda et al., 2007; Youssef et al., 2017
	Naked (free-threshing) grains	*Nud*	ERF family transcription factor gene regulating a lipid biosynthesis pathway (Transcription factor)	Taketa et al., 2008
Soybean	Determinate growth habit	*Dt2*	Plant height of semi-determinate plants is associated with GA signaling	Zhang et al., 2019a
Tomato	Fruit size	*fw2.2*	Similar to human RAS, *SlKLUH* is the causal gene for the *fw3.2* QTL and encodes a CYP450 of the 78A class	Frary et al., 2000
		*SUN*	Regulating auxin biosynthetic and responsive pathway	Xiao et al., 2008; Wang et al., 2019
Mustard	Flowering Time	*BrFLC1*	Interacts with the vernalization pathway (MADS-box transcription factor) and coordinate with gibberellic acid pathway	Yuan et al., 2009

### Transcriptional Control for Loss of Seed Shattering Trait in Cereal

From an evolutionary viewpoint, natural selection allows wild plant species to have specific functions to disperse seeds and fruits. *Although from the agronomic viewpoint, natural seed dispersal is an undesirable trait in crops as it leads to significant seed loss in harvest. Consequently, natural seed dispersal was strongly chosen against by ancient humans to ensure productive cultivation during the domestication period* (Purugganan and Fuller, 2009; Lenser and Theißen, 2013). The non-shattering traits were considered as the landmark of domestication in seed crops, as it makes the domesticated species mostly rely on human activity for propagation and enables the fixation of other domestication traits (Purugganan and Fuller, 2009). Seed crops have established their reduction of seed shattering ability independently and it is a convergent morphological adaptation to artificial selection (Purugganan and Fuller, 2009; Olsen and Wendel, 2013).

In cereal, seed shattering or fruit dehiscence is enacted through an abscission layer in the lemma-pedicel joint. Various transcription factors (TFs) coding genes were found in rice (*Oryza sativa*), which are involved in decreasing seed shattering. *Shattering4* (*Sh4*) encodes the TF with Myb3 homology and is important for the formation of a functional abscission layer in the pedicle (Li et al., 2006). *A single change of amino acid in DNA binding domain of Sh4 is intimately linked to the reduced seed shattering in domesticated rice. Also, the expression of the domesticated allele has been substantially reduced compared to the wild allele* (Li et al., 2006). *Thus, the combination of coding and regulatory alteration of* Sh4 *seems to affect the formation of the abscission layer, and consequently tries to weaken the shattering phenotype* (Li et al., 2006). *qSH1* is a major QTL on chromosome 1 involved in seed shattering in rice. The main gene, *qSH1*, codes a homeobox transcription factor-like BEL1 which is homologous to AtRPL (Konishi et al., 2006). A single nucleotide polymorphism (SNP) in the 5′-regulatory region effectively nullifies *qSH1* expression in the preliminary abscission layer in the early development stage and contributes to non-shattering traits of rice (Konishi et al., 2006). Interestingly, the regulatory SNP in the homologs of RPL promoter are also amenable for distinct structures of seed dispersal based on natural selection of Brassica species with diminished replum development (Arnaud et al., 2011). These studies show a notable convergent mechanism where the same regulatory SNP could describe developmental variations in seed dispersal structures, which are important for both domestication and natural selection in distant species (Arnaud et al., 2011; Gasser and Simon, 2011). *SH5* is another homeobox type *BEL1* gene with a high *qSH1* homology. SH5 has been expressed in the abscission layer (Yoon et al., 2014). Knockout of *SH5* inhibits abscission layer formation and prevents seed shattering. Over-expression of *SH5* leads to higher seed shattering, a consequence of decreased pedicel lignin levels (Yoon et al., 2014). The regulatory pathway of abscission layer formation has recently been expanded to include *Shattering abortion 1* (*SHAT1*), an AP2 transcription factor encoding gene (Zhou et al., 2012). *SHAT1 is needed for seed shattering by specifying abscission layer.* Sh4 *positively regulates the SHAT 1 expression in the abscission layer.* qSH1 *expression is lost in abscission layer in both the shat1 and sh4 mutant background, indicating qSH1 acts downstream of the shat1 and sh4 in the abscission layer establishment* (Zhou et al., 2012). *Intriguingly*, qSH 1 *is also needed in the abscission layer for expression of* SH1 *and* Sh4. *Thus the* qSH 1 *possibly takes part in a positive feedback loop of SH1 and Sh4 by establishing the* SHAT1 *and* Sh4 *expression in the abscission layer* (Zhou et al., 2012). *While* SH5 *and* SHAT1 *play a role in differentiating the abscission layer, the question remains whether both are artificially selected domestication genes. Like rice, decrease of seed shattering in domesticated sorghum is a result of loss of abscission in the joint that connects the seed hull with the pedicel. In sorghum, seed shattering is regulated by a single gene*, Shattering1 (Sh1), *which encodes a transcription factor YABBY. The non-shattering trait can be accounted for by any one of the three different loss-of-function mutations selected independently during sorghum domestication process* (Lin et al., 2012). *The notable mutations in* Sh1 *orthologs in rice and maize may be related to the shattering decrease in these crops* (Lin et al., 2012). Whether *Sh1* has been rewired into an SH5-directed seed shattering network in rice remains to be investigated in the future. In a wild relative of sorghum (*Sorghum propinquum*), seed shattering is conferred by the *SpWRKY* gene. It is believed that *SpWRKY* controls cell wall biosynthesis genes negatively in the abscission layer. Even so, *SpWRKY* was not crafted by artificial selection to contribute to the non-shattering characteristic for domesticated sorghum (Tang et al., 2013). These above studies together have raised a fascinating potential that the convergent domestication of non-shattering crops may have achieved the same underlying genetic goals by parallel selection (Lenser and Theißen, 2013).

In domesticated wheat (*Triticum aestivum*) free-threshing trait (loss of spike shattering tendency) is conferred by important *Q* gene (Simons et al., 2006). Q*-gene encodes the AP2-family transcription factor. The domesticated Q allele is abundantly transcribed than the wild q allele. Besides, both alleles differ in single amino acid, which significantly improves the homo-dimerization ability of the cultivated allele* (Simons et al., 2006). *Similar to Sh4, the development of the free-threshing character in cultivated wheat might also have been due to the combination of the coding and regulatory changes in the cultivated gene. The difference of expression between Q and q seems more significant as it can clarify the free threshing character in cultivated wheat* (Simons et al., 2006; Zhang et al., 2011). Even though mutation which gives rise to Q has a significant effect on the process of wheat domestication, as it helps farmers to harvest the grain more effectively, the exact cellular cause contributing to free-threshing character is still unclear. Similar research has been progressed in non-cereals crop such as overexpression *AtFUL* to make the pods shattering resistance in *Brassica juncea* (Østergaard et al., 2006).

### Cross-Talk Between Phytohormones and Related Genes Regulating Seed Shattering and Dehiscence Zones (DZ)

Hormonal homeostasis and interactions have been found recently as direct downstream effects of the core genetic network. As an example *indehiscent (IND)* expression is involved in the formation of local auxin minimum at the margin of the valve by regulating the auxin efflux in the separation layer cells (Sorefan et al., 2009). Further findings reveal that another b-HLH class SPATULA (SPT) transcription factor, required for carpel fusion early in the female reproductive organ development, may interact physically with IND (Girin et al., 2011). Auxins and cytokinins play an antagonistic role in plant growth and development (Bishopp et al., 2011). This scenario also indicates that the cytokinin signaling pathway is active at the valve margins and such a signaling pathway is interrupted in the shp1/2 and *ind* mutant. Consequently, local application of cytokinins in the fruit development can help to restore valve margin formation and further enhance dehiscence in shp1/2 and ind mutants, suggesting that cytokinins play a crucial role in valve margin differentiation (Marsch-Martínez et al., 2012). Recent studies reveal gibberellins (GAs) are also involved in the establishment of separation layer cell identity, in addition to auxins and cytokinins (Arnaud et al., 2010). As per the “relief of restraint” model, GA-mediated degradation of DELLA protein is important for GA signaling and also necessary to trigger expression of downstream genes (Harberd, 2003; Sun and Gubler, 2004). *GA3ox1*, which facilitates the final step in bioactive GAs synthesis, is shown as the direct target of IND. ALC interacts physically with DELLA repressors and local GAs production destabilizes the DELLA protein and relieves ALC to play its role in SL cell specification (Arnaud et al., 2010). In summary, these findings show that many phytohormones participate in the DZ specification and indicate that precise balance between biosynthesis and response is important. Notwithstanding the studies where the function of hormones in the development of DZ have been elucidated, very few studies about how such hormonal signals are coordinated in DZ have been carried out. One of the key challenges is to unravel the complete context of the molecular mechanisms and interactions of plant hormones underlying DZ-specification.

There are many ways for minimizing crop losses due to crop shattering ranging from conventional parental selection with minimum shattering to the screening of mutants and gene editing methods. By advancing the next-generation sequencing and the marker traits associations, many genes involved in pod dehiscence were found, and a series of mutations underlying shattering resistance in several crops and their wild relatives have been identified (Fuller and Allaby, 2009; Dong and Wang, 2015). Attempts have been made to improve shattering resistance in Brassica, which include interfering in the dehiscence process by manipulating the molecular and hormonal control pathways (Fuller and Allaby, 2009; Altpeter et al., 2016) and developing transgenic lines with pod-shattering resistance (Liljegren et al., 2000, 2004). In future, studies should focus, alongside gene-editing methods, on fine-tuning of the degree of shatter-resistance with RNA interference or the use of mutated forms of genes related to shattering in various crops.

### Key Genes Targeted for Dwarfing of Cereal to Enhance the Productivity

The plant architecture is genetically controlled by a set of genes which subsequent affect yield and productivity of crop plant species. Often, mutation or knockdown of a single gene could also lead to significant change in the overall plant growth and development, subsequently plant architecture (Spielmeyer et al., 2002). In 1960s, the agricultural transformation that increased the production of rice and wheat was via the introduction of cultivars with a genetic predisposition to a short stature due to restricted elongation of stem (Silverstone and Sun, 2000). This phenotype enabled a significant partitioning of photosynthate produced from photosynthesis to sink organs like grains (Sun and Frelich, 2011).

Currently introduction of dwarfing genes is the most important aspect deployed in modern cereal breeding. The stems of tall wheat and rice crops are not strong enough to sustain heavy grains of the high yielding cultivars, which result in significant yield losses. In addition, the proportion of assimilates partitioned in grain increases yields. Genes associated with the semi-dwarf growth of the wheat and rice cultivars have been studied. In wheat, *Reduced height* (*Rht*) gene has been identified which is shown to interfere with GA signaling transduction pathway (Peng et al., 1999). Subsequently, three research groups investigated *semi dwarf1* (*SD1*) gene from rice and found that the same hormone impair the biosynthesis (Monna et al., 2002; Sasaki et al., 2002; Spielmeyer et al., 2002). Thus, gibberellin hormone appears to be central to plant stature control.

#### Wheat *Rht* Gene and Gibberellin Signaling

The Green Revolution’s wheat dwarfing genes originated in Japan (Gale et al., 1985). The *Norin 10* dwarfing genes are now available worldwide in 70% of current commercial wheat cultivars. Norin10 contains two dwarfing genes that are semi-dominant homologous alleles on Chromosomes B and D. These alleles are labeled as *Rht-B1b* (formerly *Rht1*) and *Rht-D1b* (*Rht2*) to reflect their chromosome position (Boerner et al., 1996). The Rht alleles cause a reduced response to the plant hormone GA class (Gale et al., 1985). These plant hormones are diterpenoid carboxylic acids, that are involved in several processes of development in higher plants, including stem elongation (Hooley, 1994). The Rht gene is an ortholog of *Arabidopsis GA-Insensitive (GAI)* and maize *dwarf 8* genes, for which mutations result in GA-insensitive dwarfs (Peng et al., 1999). *Rht-1a/d8/GAI* (wild type protein) is a subgroup of the GRAS family of proteins that are thought to act as transcriptional regulators (Pysh et al., 1999). Peng et al. (1999) reported base substitutions in the *Rht-B1b* and *Rht-D1b* alleles that insert stop codons within the DELLA region. They mentioned that translational re-initiation at one of several methionines which follow the stop codon could lead to the formation of truncated Rht protein without the DELLA domain, which functions as a constituent (GA insensitive) growth repressor. The D8 (Peng et al., 1999) and GAI mutations (Peng et al., 1997) also lead to partial or complete deletion from one or both of the conserved domains. The Rht-1a/d8/GAI proteins thus function as negative GA signaling regulators and suppress GA function, provided N-terminal domains are present (Harberd et al., 1998; Dill et al., 2001). To support this concept, ectopic expression of GAI (Peng et al., 1999) in rice caused dwarfism and loss of function mutations in Rht-like genes in some cases produces an over-growth phenotype (Ikeda et al., 2001; Chandler et al., 2002). Besides *d8*, *Rht-1a* orthologs were reported in rice (known as *OsGAI* or *SLR1*) (Ogawa et al., 2000; Ikeda et al., 2001) and barley (SLN1) (Chandler et al., 2002). While cereals have a single case of Rht-1a/d8/GAI type proteins, Arabidopsis contains a gene family encoding RGA proteins and three RGA-like proteins (RGL1, -2, -3) in addition to GAI. The Arabidopsis homologues seem to overlap in their function in various GA-regulated developmental processes (Olszewski et al., 2002). It is unknown how a single protein in cereals crops is functionally equivalent to five proteins in Arabidopsis; such variation may indicate major functional redundancy in Arabidopsis or fundamental differences in GA signaling pathways between Arabidopsis and Gramineae members. Recently, some progress was made in understanding the function of Rht-like proteins and their GA repression. RGA (Dill et al., 2001), SLR1 (Itoh et al., 2002), and SLN1 (Gubler et al., 2002) are found in the nucleus and thus rapidly degraded with GA presence, the DELLA domain needed for this process. Rht’s upstream signal transduction pathway is still unknown, but GA-induced degradation is believed to involve ubiquitin-mediated proteolysis (Chandler et al., 2002).

#### Rice *sd1* Gene and Gibberellin Biosynthesis

Unlike *Rht*, the *sd1* mutation of rice is recessive and normal height can be restored in mutants using GA application showing that they have been impaired in GA production (Ashikari et al., 2002). Three research groups independently isolated the *sd1* gene and showed it encodes GA 20-oxidase (GA20ox), an enzyme involved in biosynthesis of GA (Monna et al., 2002; Sasaki et al., 2002; Spielmeyer et al., 2002). Two of these research groups have used positional cloning to detect a GA20ox open reading frame close to the sd1 locus on the long chromosome arm (Monna et al., 2002; Spielmeyer et al., 2002). *They also reported mutations in corresponding genes from semi-dwarf varieties. The third group, which had inferred the gene’s identity by the effect of GA content mutations, used PCR to amplify DNA fragments, corresponding to two GA20ox genes, one of which mapped to the sd1 loci* (Sasaki et al., 2002; Ashikari et al., 2002). Semi-dwarf rice cultivars with Dee-geo-woo-gen sd1 allele contain a 383-bp deletion in the *GA20ox* gene (known as *OsGA20ox2*), which incorporates stop codon that is likely to result in a highly truncated, inactive enzyme. Gibberellin 20-oxidases are 2-oxoglutarate-dependent dioxygenases catalyzing carbon-20 depletion in the penultimate stage in biosynthesis of GA (Hedden and Phillips, 2000). These oxidases are encoded by small gene families, members of which have partial functional redundancy due to overlapping (but different) expression profiles or because of movement of the intermediates synthesized by enzymes between tissues. Therefore, loss-of-function GA20ox mutants are relatively less GA-deficient and are semi-dwarfs, unlike significant GA-deficient plants, which are extremely dwarfed and sometimes sterile. Two *GA20ox* genes were defined in rice: *OsGA20ox1* (Toyomasu et al., 1997) and *OsGA20ox2*. Remarkably, selection for semi-dwarfism in rice has consistently yielded mutations in *OsGA20ox2* instead of *OsGA20ox1* or another GA-biosynthesis gene (for example, *GA 3-oxidase* is also encoded by a multi-gene family). Mutations in other genes might have a severe developmental impact or have negative impact on yield, and thus have been not selected in breeding programs. Genetic and functional analyses of *SLR1/RHT* and *SD1* genes in rice and wheat have enormously improved the understanding of GA biosynthesis and signals, resulting in a strong methodology for manipulating the plant height of major crops. Both cases illustrate the central role played by GAs in controlling developmental processes. Therefore, GA signaling pathways (biosynthesis and signal transduction) are key aspects for manipulation in pursuit of further crop yield improvements. The yields of existing cereal crops seem to be approaching their limit, and new interventions are required if population is not to outstrip the food supply. Targeted genetic engineering/modification using newly emerged genomics, genome-editing technologies may be part of the next Green Revolution.

### Achieving Submergence Tolerance

The incidences of uncertain rain and flood have been increased due to continued climate change. Today, more than 30 percent of the rice-planting land is vulnerable to flooding resulting in crop loss. In 1960s, the development of semi-dwarf variety was one of greatest achievement which significantly addressed the issue of global hunger threat caused due to human population explosion. The suppression of GAs production in the stem reportedly made high yielding semi-dwarf rice varieties susceptible to one of the most important abiotic stress “water logging.” These developed semi-dwarf rice varieties lacked submergence tolerance. The lower nodes of these varieties unable to produce enough gibberellins to trigger elongation of the internode.

#### Genomics Based Discovery of Genomic Regions Associated With Submergence Tolerance

Submergence stress causes several adverse impacts on a plant such as low light intensity, hypoxia, nutrient effusion, physical injury, susceptibility to pathogen and pests attacks (Angaji et al., 2010). Several QTL mapping studies reported number of QTLs controlling submergence tolerance (Xu and Mackill, 1996; Nandi et al., 1997; Toojinda et al., 2003). A major QTL (Sub1) for submergence tolerance has been identified on chromosome 9 with LOD 36 and 69% of phenotypic variance explained (PVE) (Xu and Mackill, 1996). Sequencing of Sub1 genomic region identified three genes which encodes a ERFs (Sub1A, Sub1B, and Sub1C) in which Sub1A has been reported as a key component of submergence tolerance (Xu et al., 2006). Further cloning and characterization of Sub1 QTL helping in the detection of responsible genes and also help to discover tightly linked gene-based markers for molecular breeding program (Siangliw et al., 2003; Toojinda et al., 2005; Neeraja et al., 2007). Furthermore, in other studies major QTLs namely qAG9-2 on L.G. 9 and qAG7-1 on L.G. 7 were reported (Angaji et al., 2010; Septiningsih et al., 2013). Later on, qAG9-2 QTL has been fine mapped and found a candidate gene OsTPP7 which encodes a trehalose-6-phosphate phosphatase which is responsible to regulate anaerobic generation (Kretzschmar et al., 2015). Both Sub1 and qAG9-2 major QTLs are widely used in rice breeding programs to improve submergence tolerance at germination and vegetative stages. Utilizing genomics resources several breeding efforts are also made in developing submergence tolerance varieties to sustain rice production. Various landraces and traditional genotypes namely, Kurkaruppan, FR13A, Thavalu, Goda Heenati, etc., were reported to be a suitable source of alleles which is associated with submergence tolerance (Miro and Ismail, 2013).

#### Precise Characterization of Genes Governing Submergence Tolerance

In recent years significant progressed have been made toward understanding the physiological, biochemical and genetic basis of submergence tolerance, to identify the causal gene(s) that are crucial for submergence tolerance (Oladosu et al., 2020). Recently, Kuroha et al. (2018) identified the gene *SD1* (*SEMIDWARF*) responsible for submergence-induced elongation of internode by producing gibberellins mainly GA4. Another study identified genes *SNORKEL 1 (SK1)* and *SK2* which encodes for ERFs, appeared to trigger submergence tolerance via ethylene signaling (Hattori et al., 2009). Both gene products further facilitate the internode elongation through GAs. Previous study identified a submergence tolerance gene *SUB1A* (an *Ethylene-response-factor-like* gene) on chromosome 9 which encodes ERFs (Xu et al., 2006; Fukao et al., 2006). During flash floods, *SUB1A* inhibits plant elongation at the seedling stage. Flash floods usually last for a few weeks. Cultivars carrying *SUB1A* tolerance gene show stunted growth and can survive in submerged conditions for a few weeks. Both *SNORKEL 1* and *SNORKEL 2* (*SK1/2*) genes and *SUB1A* encode ERFs which are associated with GAs, but they act in opposite ways in controlling plant development in response to submergence. Further more research is required to uncover the various pathways associated with *SK1*; *SK2* and *SUB1A*. Furthermore, recently two genes have been identified *ACCELERATOR OF INTERNODE ELONGATION 1* (*ACE1*) and *DECELERATOR OF INTERNODE ELONGATION 1* (*DEC1*) which are responsible to control stem elongation (Nagai et al., 2020). *ACE1* gene encoding an unknown function protein which is associated with internodes elongation via GAs, whereas, *DEC1* gene encoding a zinc –finger TF, which suppresses internodes elongation. Both the genes influence gibberellin-activated cell division in stem nodes. The expression of *ACE1* gene during submergence conditions in rice triggers elongation of internodes within a cell-division zone of the plant. This results in an increased number of elongated internodes and increased plant height. Further gene *ACE1C9285* is controlled by SUB1C, a gibberellin-activated TF which is upregulated in response to submergence (Fukao and Bailey-Serres, 2008). *SUB1C* expression level seemingly low in cultivars that contain the *SUB1A-1* regulator gene, a homolog to *SUB1C*. In short rice cultivars expressing gene *SUB1A-1*, GAs responsiveness altered, subsequently use carbon pool for leaves elongation, and restrict overall plant development and enter to transient quiescent stage during flooding, an adaptation to overcome deep floods (Fukao et al., 2006; Xu et al., 2006). In semi-dwarf cultivars, internodes elongation only takes place in the upper internodes during growth stage. Nagai et al. (2020) reported a gene *ACE1-LIKE1*, which triggers upper internodes growth in deep-water. Presently, these omics study based information on the genetic basis of submergence tolerance is the base of rapid improvement of plant architecture to design a high yielding crop tolerant submergence.

## Translation of Omics Driven Data for Re-Domestication and *De Novo* Domestication: Utilization of Genome/Gene Editing Tool

**FIGURE 2:**
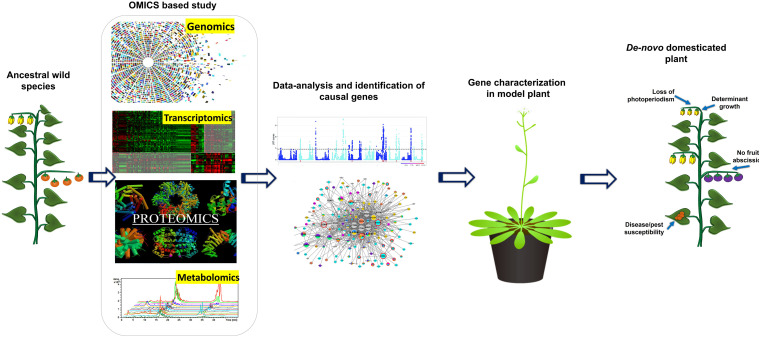
Schematic diagram representing the role of OMICS based research in gene characterization and development of designer crops using *de novo* domesticated crops approach.

Gene-editing technologies have become choice of a researcher to domesticate neglected crops and wild relatives in a short period (Fernie and Yan, 2019). Traditionally, plant domestication and the development of productive cultivars required decades of breeding, which is also the key reason why so many breeding programs over the last 100 years focused on further improvement of a relatively small number of crops. Recent identification of several major domestication genes and scientific breakthroughs in integrating various genomic changes in plants concurrently with CRISPR/Cas9 editing has allowed re-domestication of existing crop plants and *de-novo* domestication wild species to be domesticated within a single generation (Figure 2) (Schindele et al., 2020). *De-novo* domestication has contributed to agro-biodiversity and diet quality, with possible future environmental and nutritional benefits (Singh et al., 2019). In the history of crop domestication amid higher yield selection and breeding, international germplasm exchange; multiple local resistance and resilience genes of wild species have been lost or have never been completely incorporated into breeding lines (Fernie and Yan, 2019). In other words, wild relatives of domesticated plants have significantly higher variable gene pool than that of domesticated ones (Hickey et al., 2019). As we start to uncover more about the framework of crop genomes and the loci of quality traits, there are chances of incorporating valuable characters into existing crop species and ways to quickly re-domesticate new crops. This step can be effectively achieved using breakthrough CRISPR-Cas9 gene-editing technologies, in particular, to introduce beneficial alleles without linkage drag (Li et al., 2018), to produce novel quantitative variations (Rodríguez-Leal et al., 2017), direct deletion of deleterious alleles (Johnsson et al., 2019), and/or higher recombination rates (Mieulet et al., 2018). Recently, gene editing has been shown to enhance plant architecture, flower development, and fruit size in *Physalis pruinosa* (Lemmon et al., 2018). Gene editing is a promising method to generate diversity and to compensate for the genetic hitchhiking effects in germplasm. For reference, associated selection of traits such as fruit weight and disease resistance altered the tomato metabolome, providing an opportunity for precise breeding to alter nutritional and flavor traits (Zhu et al., 2018). These hitchhiking effects and others, such as those found in rice and maize, represent promising goals for genetic modification to fettle linkage drag (Palaisa et al., 2004). For instance, African rice landrace Kabre possess superior resistance to pests and tolerance to drought; however, during domestication the plant architecture compromised affecting their overall yield potential. To address this Lacchini et al. (2020) targeted multiples genes which control plant architecture (*HTD1*) and control seed size and/or yield (*GS3, GW2*, and *GN1A*) by generating knockouts through multiplex CRISPR/Cas9. In knockouts, mutation in *HTD1* gene caused reduced plant high to diminish lodging and improved tillering, whereas mutations in *GS3, GW2*, and *GN1A* resulted increased panicle and length along with improved seed girth. Earlier, Hu et al. (2019) demonstrated generation of semi-dwarf rice lines by targeting gene *SD1* and *Photosensitivity5* (*SE5)* in elite landraces Kasalath. In this post genomics, the technique CRISPR/Cas has received overwhelming response and till dates several knockouts of rice elite varieties are available with improved traits by targeting specific genes which were characterized due to viability of several omics approached era. Some of the examples for the targeted traits and gene targets in rice are *LAZY1* for tiller-spreading, *Gn1a, GS3*, and *DEP1* for improved grain number, size and dense erect panicles, *SBEIIb* for High amylose content, *OsERF922* for enhanced blast resistance, *OsSEC3A* for resistance against blast causing pathogen *Magnaporthe oryzae, OsSWEET13 for* bacterial blight resistance, *ALS* and *EPSPS* for herbicide resistance, *OsPDS, OsMPK2, OsMPK5, OsBADH2, OsAOX1a, OsAOX1b, OsAOX1c*, and *OsBEL* for tolerance against various abiotic stress, OsHAK-1 for low cesium accumulation, and *OsPRX2* for potassium deficiency tolerance (Shan et al., 2013; Xie and Yang, 2013; Shan et al., 2014; Xu et al., 2014; Zhang H. et al., 2014; Zhou et al., 2014; Woo et al., 2015; Meng et al., 2017; Nieves-Cordones et al., 2017; Mao et al., 2018; Ma et al., 2018). Likewise, in wheat *EDR1*, *TaMLOA1, TaMLOB1*, and *TaMLOD1* targeted for resistance to powdery mildew, and *GW2* and *TaGW2* targeted for increased grain size, weight and protein content (Shan et al., 2014; Wang et al., 2014; Gil-Humanes et al., 2017; Kim et al., 2018; Wang et al., 2018). In orphan crops cassava and flax herbicide resistance has been introduced by targeting a gene *EPSPS* (Sauer et al., 2016; Hummel et al., 2018); whereas *ALS* was targeted in soybean (Cai et al., 2015). Similarly, many traits have been introduced or improved by targeting various genes in some economically important crops plants such as maize, tomato, potato, grapes, orange, cucumber, tea, etc. (Adhikari and Poudel, 2020; Bhatta and Malla, 2020).

**FIGURE 3:**
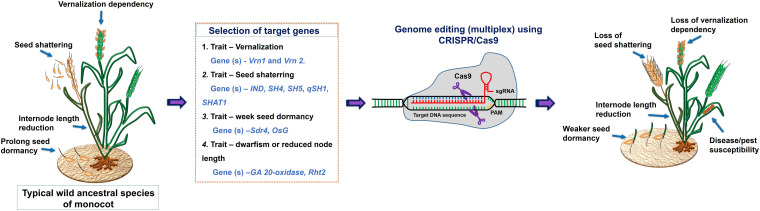
A schematic representation of a draft model for the selection of target genes for CRISPR/Cas9 mediated domestication of wild ancestral species of monocot.

The wild ancestral species of crop plants such as *Solanum pimpinellifolium* for tomato; *Solanum demissum* and *S. stoloniferum* of potato; *Fragaria vesca* of strawberry; *Teosinte* and *Tripsacum* of maize; *Triticum dicoccoides*, and *T. turgidum L.* ssp. *Durum* of wheat; *Oryza rufipogon* and *O. longistaminata* of rice*; Manihot glaziovii* and *M. neosana* and *Glycine soja* of soybean have been used for introgression key agronomic important traits into cultivars though breeding program (Zsögön et al., 2017). Moreover, most of the domesticated related traits and associated genes well characterized and has been linked with the metabolic pathway(s), and/or hormone biosynthesis and signaling (Table 4); therefore, integrated omics approach which also involved metabolomics study has provided insights into the molecular basis of trait domestication. One can target these domesticated genes in wild ancestral plants for their speedy domestication. Now through CRISPR-Cas9 method these wild relative can be directly used for re-domestication or *de-novo* domestication (Figure 3 and Tables 5, 6). One of the important case study of *de novo* domestication in tomato has been done by Zsögön et al. (2018) by targeting important domestication related genes through CRISPR-Cas9 in tomato wild ancestral species *S. pimpinellifolium*. Zsögön et al. (2018) targeted *SELFPRUNING* (*SP*, control general plant growth habit), *OVATE* (*O*, regulate fruit shape); *FASCIATED* (*FAS*), *FRUIT WEIGHT* 2.2 and *CLAVATA3* (*CLV3*) (control fruit size and locule numbers), *MULTIFLORA* (*MULT*, regulate fruit number), and *LYCOPENE BETA CYCLASE* (*CycB*). The engineered *S. pimpinellifolium* lines and achieved remarkable change in the plant overall phenotype with important traits essential for the commercial purpose such as increased lycopene content, enhanced fruit shape and determinant growth of plant; moreover, this was achieved in just single generation. Another study involved editing of multiples genes *SP, SP5G* (control day-length insensitivity), *CLV3*, WUSCHEL (*WUS*) and *GDP-L-galactose phosphorylase 1* (*GGP1*, control biosynthesis of ascorbic acid) in *S. pimpinellifolium* (Li et al., 2018). This study clearly showed how selective editing of domesticated related genes can completely alter the plant architecture and improves the nutritional quality of fruits and makes convert wild relative into domesticated crop with retained biotic and abiotic stress tolerance properties (Li et al., 2018). Very recently, in the wild strawberry (*Fragaria vesca*) few attempts has been made to demonstrate the procedure of the re-domestication or *de novo* domestication (Zhou et al., 2018; Feng et al., 2019). These attempts involved editing of genes *tryptophan aminotransferase of Arabidopsis 1* (*TAA1*, converts tryptophan to indole-3-pyruvic acid), *Auxin response factor* 8 (*ARF8, repressor of auxin signaling*) and *YUCCA10* (*YUC10*, family of flavin-containing monooxygenases convert IPyA to IAA), key auxin biosynthetic and signaling pathways genes. Rice has five allotetraploids (BBCC, CCDD, HHJJ, HHKK, and KKLL) wild species which are also valuable genetic resources for improving of elite rice varieties. Among them the CCDD (species from South America genome) possess much stronger biotic and abiotic resistance and larger biomass compared to the cultivated diploid rice. Recently Yu et al. (2021) demonstrated *de novo* domestication of wild allotetraploid rice PPR1 (*O. alta*; CCDD type genome) by improving six agronomically important traits *viz* nutrition use efficiency, abiotic stress tolerance, grain yield and quality, heading date, biotic stress resistance and sterility by genome editing targeting multiple genes including *OaSD1-CC, OaSD1-DD, OaAn-1-CC*, and *OaAn-1-DD* by CRISPR/Cas9 method. This suggests that CRISPR/Cas is a promising approach tool for the domestication of crops (Crews and Cattani, 2018), and is highly important for characters of defined selective sweeps in related species. These achievements were possible due to precise prediction of causal genes and metabolic pathways achieved by interpretation of data generated through genomics, transcriptomics, metabolomics, etc.

**TABLE 5:** List of genes targeted in wild ancestral species of tomato and strawberry to demonstrate *de novo* domestication.

Wild relative	Target Gene	Traits modification	References
*Solanum pimpinellifolium*	*CLV3, WUS, SP, SP5G, and GGP1*	Plant height and response to phtotoperiodism, flower numbers, and fruit size and shape, and ascorbic acid content	Zsögön et al., 2018
	*OVATE, MULT, FAS, SP, and CycB*	Plant architecture and habitat, flower numbers, and fruit size and shape, and lycopene content	Li et al., 2018
*Fragaria vesca*	*FveTAR1* and Fve*YUC10*	Auxin biosynthetic and signaling genes affecting plant growth and reproductive organ development	Feng et al., 2019
	*FveTAA1* and *FveARF 8*	Auxin biosynthetic and signaling genes affecting plant growth and reproductive organ development	Zhou et al., 2018

**TABLE 6:** A model representing state of art for selecting the genes which can be edited to domesticate crop wild ancestral species through CRISPR/Cas9 approach.

Crop Name	Target Gene	Function	References
*Zea Mays*	*Tb1*	TCP-gene family TF which is involved in suppression of side branching changes the source/sink relationships; yields increase.	Doebley et al., 1997; Studer et al., 2011
	*tga1*	SBP-box TF have a key role in alteration of the encased kernel to naked kernel	Wang et al., 2015
	*CCT*	CCT domain-containing protein gene involved in decrease of photoperiod sensitivity	Yang et al., 2013; Huang et al., 2018
*Glycine max*	*DT1*	CETS is a family of regulatory genes which are involved in transforming indeterminate growth to determinate, resulting in developing a compact crop.	Tian et al., 2010; Cai et al., 2018
	*GA20ox*	Key enzyme involved in Gibberellin biosynthesis and identified as its association with seed weight	Lu et al., 2016
	*SHAT1-5*	Plant specific NAC gene family TF involved in the biosynthesis of secondary cell wall which facilitating fiber cell cap thickening result in a decreasing the rate of pod shattering	Dong Y. et al., 2014
*Solanum lycopersicum*	*ARF19*	Auxin response factor 19 TF reported being a negative regulator of fruit set	De Jong et al., 2009
	*BRC1a*	*BRANCHED1a* gene encoding a TCP family TF which involved in the regulation of lateral shoot outgrowth	Martín-Trillo et al., 2011
	*CHI*	Chalcone Isomerase is associated with flavonoid biosynthesis	Willits et al., 2005
	*S*	*Compound inflorescence (s)* encodes a homeobox TF which controls the number of flower/fruits per inflorescence architecture	Lippman et al., 2008
	*CKX*	Cytokinin oxidase enzyme associated gene is involved in the inactivation of bioactive cytokinin	Ashikari et al., 2005
	*FAS*	*CLAVATA3* encoded the *Fasciated* gene which is associated with controlling locules number and size in fruit	Xu C. et al., 2015
	*GLK2*	Golden2-like TF belongs to GARP family which play a key role in the regulation of chloroplast development in fruits	Powell et al., 2012
	*J1*	*JOINTLESS* belongs to MADS-box gene family controlling the development of the abscission zone in pedicels	Mao et al., 2000
	*Cyc-B*	Lycopene β-cyclase involved in the catalyzes the conversion of lycopene into β-carotene	Ronen et al., 2000
	*NOR*	*Non-ripening* gene associated with the initiation of the normal fruit ripening	Seymour et al., 2013
	*O*	*OVATE* is a regulatory gene involved in the regulation of fruit shape	Liu et al., 2002
	*PRO*	*PROCERA* gene involved in suppression of gibberellin signaling	Jasinski et al., 2008
	*RIN*	*RIPENING INHIBITOR* gene belongs MADS-box family; key role in controlling biosynthesis of ripening -related ethylene	Seymour et al., 2013
	*SP*	*SELF-PRUNING* gene is a developmental regulator associated with indeterminate and sympodial growth habit in tomato	Pnueli et al., 1998
	*SFT*	*SINGLE FLOWER TRUSS* gene involved in regulation of flowering	Lifschitz et al., 2006
	*CLV3*	*CLAVATA3* key meristematic gene, regulating locule numbers in fruit	Rodríguez-Leal et al., 2017
	*PSY1*	Phytoene synthase 1 gene involved in the biosynthesis of carotenoid resulting in yellow flesh fruit	Hayut et al., 2017
	*ANT1*	*Anthocyanin mutant 1* gene encodes a *Myb* TF which involve in increasing anthocyanin content	Čermák et al., 2015
	*GAD2, GAD3*	Key genes encoding an enzyme glutamate decarboxylase for biosynthesis of γ-aminobutyric acid (GABA) in fruit	Nonaka et al., 2017
	*ALMT9*	*Al-ACTIVATED MALATE TRANSPORTER9* gene involved in decreasing the malate content accumulation in fruit	Ye et al., 2017
	*MBP21*	*MBP21* is a MADS-box protein controlling formation of abscission zone in pedicel	Roldan et al., 2017
	*BOP1, BOP2, BOP3*	*BLADE ON PETIOLE* gene reported being associated with early flowering with simplified inflorescences	Xu et al., 2016
	*SP5G*	*SELF-PRUNING 5G* gene is a flowering repressor linked involved in the development of day-length-sensitive tomato plant	Soyk et al., 2017
*Cucumis sativus*	*WIP1*	*WIP1* is a C2H2 zinc finger TF gene involved in development of gynoecious plant	Hu et al., 2017
*Actinidia chinensis*	*CEN*	*CENTRORADIALIS* like gene associated with the development of compact plant with early terminal flowering and fruit development	Varkonyi-Gasic et al., 2019

## Conclusion

Omics have helped plant biologists to dissect important developmental clues and gene characterization. Presently, multidimensional omics approach where the biological sample can be analyzed for transcriptomics, proteomics and metabolomics in parallel, etc; offers plant biologists a complete understanding of plant metabolism by revisiting the metabolic pathways or identification of newer pathways. In the past 20 years, plant biologists have gathered significant amount of data relevant to genomes, transcriptome, proteome, and metabolome. Recent attempts are on development of gene-expression and proteome atlas. Altogether, this would strengthen the knowledge of the metabolic pathways, which have played crucial role during domestication of crop as well as trait improvement. Now, this knowledge has been translated to develop designer crops with desired traits by editing metabolic pathways of wild ancestral species (rich resource of genetic variations) called as *de novo*-crop domestication. Domestication of wild or semi domesticated crop (tolerant to stress responses) would be feasible by multi step process were few important traits need to be improved first using genome editing; later the homologous lines can be selected for next level of trait modification. Such approach would be able to deliver a commercial line in 5 to 10 years. The CRISPR/Cas technique need to be explored in full extent by targeting several traits such as bio-fortification of nutrition’s; because the current growing population also demand nutritional security. To achieve this, analysis of resequencing data available for the several crops is important; including GWAS which can identify high quality SNPs and haplotypes associated with target trait. Therefore, we expected in next 20 years’ omics technology driven *de-novo* crop domestication will play very important role in the field of plant biotechnology.

## Author Contributions

RK received the invitation and conceived the plan for the manuscript. RK and VS wrote the manuscript. AK, SS, DR, SK, KP, BH, AV, RK, MP, ST, and GN improved the section and developed the table and figures. ST helped in developing the revised version. All the authors have read the manuscript before submission.

## Conflict of Interest

The authors declare that the research was conducted in the absence of any commercial or financial relationships that could be construed as a potential conflict of interest.
